# Noise Generation After Total Knee Arthroplasty: Comparison Between Mobile-Bearing and Cruciate-Retaining Designs with Minimum Two-Year Follow-Up

**DOI:** 10.3390/jcm15176898

**Published:** 2026-09-06

**Authors:** Jérôme Murgier, Thibaut Tourcher, Sonja Cabarkapa

**Affiliations:** 1 Aguilera Private, Clinic Ramsay Santé, 21 Rue de l’Estagnas, 64200 Biarritz, France; 2 Orthopaedica Department, Toulouse University Hospital, 31000 Toulouse, France; 3 St. Vincent’s Hospital, Melbourne, VIC 3065, Australia

**Keywords:** total knee arthroplasty, prosthetic noise, patient satisfaction, patient-reported outcomes, implant design

## Abstract

**Background**: The occurrence of noise after total knee arthroplasty (TKA), whether audible or perceived by the patient, is commonly reported during daily activities. Although generally benign and rarely associated with implant failure, such noise may negatively influence patient perception of surgical success and overall satisfaction. This study aimed to evaluate whether implant design affects the occurrence of prosthetic noise and to assess its relationship with patient satisfaction. **Methods**: A retrospective comparative study was conducted at a single orthopedic center, including patients who underwent primary TKA between 2020 and 2023 with a minimum follow-up of two years. Functional outcomes were assessed using the Simple Knee Value (SKV) and the International Knee Score (IKS). Prosthetic noise was evaluated using a validated questionnaire addressing noise perception during daily activities. Two implant designs were compared: mobile-bearing and cruciate-retaining prostheses. **Results**: A total of 212 patients were included. In the mobile-bearing group, 61.4% of patients reported no prosthetic noise compared with 62.1% in the cruciate-retaining group. Overall, 39% of patients perceived some degree of noise. Patients without perceived noise reported significantly higher satisfaction than those with noise, while functional outcomes (SKV and IKS) were comparable between groups. **Conclusions**: Within the limitations of this retrospective study, no statistically significant difference in prosthetic noise was detected between mobile-bearing and cruciate-retaining total knee arthroplasty designs. Prosthetic noise was associated with lower patient satisfaction but not with functional outcomes. These findings should be confirmed in adequately powered prospective studies. **Level of evidence**: III, retrospective comparative study.

## 1. Introduction

Total knee arthroplasty (TKA) is one of the most successful procedures in orthopedic surgery and is the standard treatment for advanced knee osteoarthritis when conservative management has failed [1]. In recent decades, the number of TKAs performed worldwide has steadily increased, driven by an aging population and advances in surgical outcomes [2,3,4]. Various clinical studies have demonstrated that TKA consistently ameliorates pain, restores joint function, and improves quality of life for most patients [5,6]. However, patient dissatisfaction and postoperative complications pose ongoing issues [7], including noise occurrence, such as audible or perceived popping, squeaking or clicking sounds emanating from the replaced joint. While often regarded as harmless, such noises can cause distress, diminished patient satisfaction, and can sometimes raise concerns about implant function or longevity [8]. This common post-TKA symptom has not been fully addressed by the orthopedic community [9] and there is a paucity of literature pertaining to this subject.

Mechanisms explaining noise generation in TKA are often multifactorial [10]. Factors such as surgical technique, material properties, implant design and patient-specific biomechanics are all likely contributors [11]. Despite its clinical relevance, the limited evidence and lack of management protocols available make it difficult for clinicians to address the noise [12,13]. The evidence does suggest that noise generated post-TKA appears to have a minimal impact on joint awareness and patient-reported clinical outcomes [14,15]. It is likely that prosthetic design plays a role in the presence or absence of noise, and the degree to which it proves inconvenient, with a tendency for less noise to be associated with prostheses without a cam [10]. There is some evidence to suggest that cruciate-retaining protheses confer less noise, in comparison to posterior cruciate substituting, mobile-bearing or isolated posterior cruciate ligament (PCL)-retaining [16,17]. Beyond implant survivorship, functional outcomes, and complication rates, patient-reported perception has become an essential component in evaluating the success of TKA. Among these factors, prosthetic noise has attracted increasing interest, as it may negatively impact patient satisfaction despite otherwise favorable clinical results. Mobile-bearing (MB) and cruciate-retaining (CR) designs are widely used due to their reliable outcomes and comparable long-term survival [18]. Nevertheless, their differing biomechanical principles may influence the occurrence of noise during daily activities. To date, the potential difference in noise generation between these two commonly implanted designs remains insufficiently investigated, warranting further study. As both designs show similar survival rates, implant-related noise may represent a relevant criterion when choosing between them.

The primary objective of this study was to compare the frequency of prosthetic noise between mobile-bearing and cruciate-retaining total knee arthroplasty designs. The primary outcome measure was patient-reported prosthetic noise. Secondary objectives were to evaluate the relationship between prosthetic noise and (1) functional outcomes measured by the Simple Knee Value (SKV) and International Knee Score (IKS), (2) patient satisfaction, and (3) factors associated with prosthetic noise occurrence.

We hypothesized that there would be no significant difference in noise generation between the two implant designs, but that the presence of noise may negatively influence patient satisfaction despite similar functional outcomes.

## 2. Materials and Method

**Figure 1:**
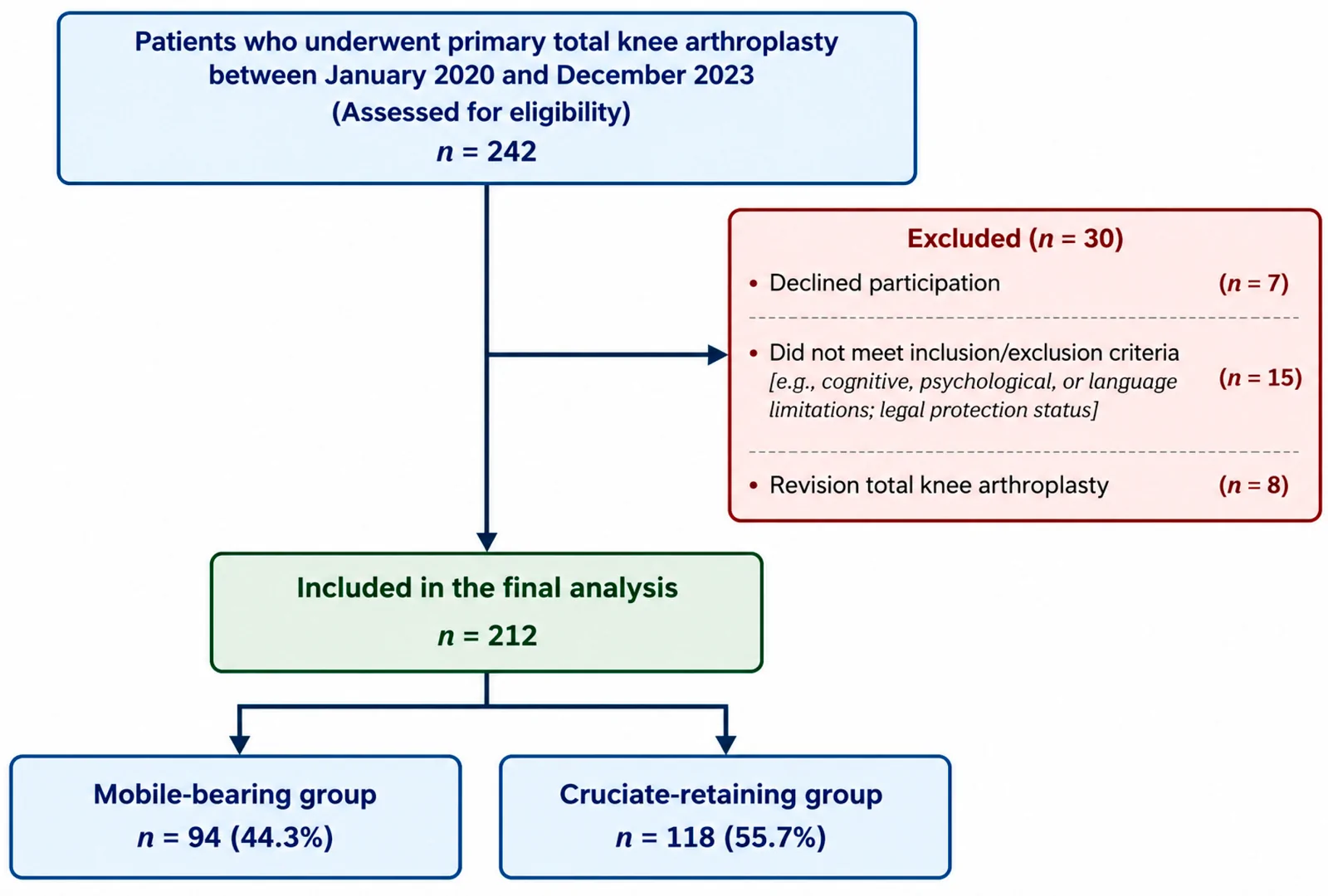
Flowchart of patient selection.

Patients who underwent primary total knee arthroplasty (TKA) with a minimum follow-up of two years in the same orthopedic department (Aguiléra private Clinic, Biarritz, France) were included in this retrospective, comparative, continuous study. All procedures were performed between 2020 and 2023 by two surgeons using a standardized mechanical alignment technique (gap balancing technique [19]) and consistent surgical and postoperative protocols. The indication for surgery was end-stage osteoarthritis in all cases, and no patellar resurfacing was performed. Depending solely on implant availability, two prosthetic designs were used: a mobile-bearing implant (SCORE® MB; Amplitude, Valence, France) and a cruciate-retaining implant (Triathlon® CR; Stryker, Mahwah, NJ, USA). The MB features a mobile tibial insert allowing rotational movement in the axial plane, aiming to reproduce more physiological kinematics and improve patellofemoral tracking. In contrast, the CR preserves the posterior cruciate ligament (PCL), contributing to enhanced knee stability and more natural joint mechanics. Exclusion criteria included revision TKA for any reason, inability to provide informed responses due to linguistic, cognitive, or psychological limitations, and patients under legal protection or deprived of liberty. Patients were contacted by telephone at a minimum follow-up of two years postoperatively. The investigator administering the postoperative telephone questionnaire was blinded to the implant design throughout data collection. All interviews were conducted using the same standardized, validated questionnaire. Among the 242 eligible patients, 212 met the inclusion criteria and were included in the final analysis: 94 in the MB group and 118 in the CR group. The mean follow-up was 32 ± 6 months. Patients excluded either did not meet the inclusion criteria or declined participation (Figure 1). The two groups were comparable in terms of age and sex distribution (Table 1). The study population comprised 116 men and 96 women, with a mean age of 68.3 ± 8.3 years.

Functional outcomes were assessed using the Simple Knee Value (SKV), which has been shown to correlate with commonly used functional scores [20,21].

Patient satisfaction was evaluated using a 5-point Likert scale (very satisfied, satisfied, moderately satisfied, not very satisfied, not satisfied at all). Prosthetic noise was assessed using the validated questionnaire on knee noise described by Kuriyama et al. [22]. Patients were asked whether they perceived noise originating from the replaced knee during daily activities. For the present study, responses were dichotomized into two categories: “silent” (no perceived prosthetic noise) and “noisy” (any reported perception of prosthetic noise, regardless of frequency or intensity). This classification was used for all subsequent analyses comparing patient satisfaction and functional outcomes.

### 2.1. Statistical Analysis

Statistical analyses were performed using SAS software (version 9.4^®^, SAS Institute, Cary, NC, USA). Quantitative variables were described using means and standard deviations (SD) or medians and interquartile ranges (IQR), according to data distribution. Qualitative variables were summarized as frequencies and percentages. Comparisons between the mobile-bearing and cruciate-retaining groups were performed using analysis of variance (ANOVA) for continuous variables and the Chi-square test or Fisher’s exact test, as appropriate, for categorical variables. Comparisons between patients reporting prosthetic noise and those reporting no noise were performed using the same statistical methods. To explore factors associated with prosthetic noise, a univariable logistic regression analysis was performed to assess the association between SKV score and prosthetic noise. Results were expressed as odds ratios (ORs) with 95% confidence intervals (CIs). A two-sided significance level of *p* < 0.05 was considered statistically significant. Given the exploratory nature of the study and the limited number of predefined outcomes, no adjustment for multiple comparisons was performed.

### 2.2. Ethical Approval

This study was conducted in accordance with the Declaration of Helsinki and approved by the French National Ethics Committee (N° ID-RCB: 2023-A00626-39).

## 3. Results

A total of 212 patients were included: 94 in the mobile-bearing (MB) group and 118 in the cruciate-retaining (CR) group. The overall rate of patients reporting prosthetic noise was 39%. In the MB group, 61.4% of patients reported no prosthetic noise compared with 62.1% in the CR group, with no statistically significant difference between implant designs. Functional outcomes were comparable between implant groups. The mean SKV score was 81.5 ± 13.2 in the MB group and 77.4 ± 15.5 in the CR group (*p* = 0.41). Similarly, the mean IKS function score was 35.6 ± 6.6 in the MB group and 34.6 ± 6.8 in the CR group (*p* = 0.69) (Table 2). Patient satisfaction was also similar between implant designs.

### 3.1. Association Between Prosthetic Noise and Clinical Outcomes

Patients without prosthetic noise reported significantly higher satisfaction than those experiencing noise. Overall, 93% of patients in the silent group were satisfied or very satisfied compared with 84% in the noisy group (*p* = 0.02). Across the entire cohort, 68% of patients were very satisfied, 24% were satisfied, 4% were moderately satisfied, 2% were not very satisfied, and 2% were not satisfied at all. The overall mean SKV score was 79.3 ± 15.3. Patients without prosthetic noise demonstrated higher SKV scores than those reporting noise (81.8 ± 14.7 versus 75.0 ± 14.6, respectively). In contrast, no significant difference in IKS function score was observed between the silent and noisy groups (34.6 ± 3.4 versus 36.0 ± 2.5; *p* = 0.12). The overall mean IKS function score was 35.2 ± 6.7.

### 3.2. Factors Associated with Prosthetic Noise

Univariable logistic regression analysis demonstrated a significant association between SKV score and the presence of prosthetic noise (OR = 0.963, 95% CI 0.936–0.992; *p* = 0.011). Each one-point increase in SKV score was associated with an approximately 4% reduction in the likelihood of reporting prosthetic noise.

## 4. Discussion

The primary finding of this study was that both prosthetic designs demonstrated comparable rates of noise generation, thereby supporting the initial hypothesis. Despite its clinical relevance, the literature addressing noise generation following total knee arthroplasty remains limited. In the present study, the overall rate of noise production was lower than that reported in studies evaluating posterior-stabilized (PS) prostheses, where more than 60% of patients reported prosthetic noise [12,16,17].

Lizcano et al. [23] evaluated prosthesis noise based on implant design by comparing ultra congruent (UC) prostheses to PS. They found that the PS group was noisier than the UC group but that it did not significantly impact patient satisfaction. However, PROMS were affected by the noise, and patients who had a noisy prosthesis had conferred lower results on PROMS. Similarly, Nam et al. [9] reported that the likelihood of noise generation was greater in PS (41%), rotating platform (45%) and gender-specific implants (36%) than in cruciate-retaining knees (23%). Pritchett et al. [16] assessed bi-cruciate-retaining prostheses with only a 4% rate of noise production, the smallest percentage published to date. This can be explained by the stability conferred by the preservation of the two cruciate ligaments and the very natural biomechanical function of the prosthesis. The increased noise in mobile-bearing and cruciate-retaining designs could potentially be explained by their respective mechanical configurations [24]. Mobile-bearing designs allow independent rotation of the tibial insert, which may lead to additional noise due to friction between the insert and the tibial tray, notably if there is a malalignment or wear [25]. Similarly, cruciate-retaining implants preserve the posterior cruciate ligament and produce more complex motion patterns, which can also contribute to noise generation [26].

Mechanisms of noise generation after TKA remain poorly understood [13], although several potential causes have been identified. Noise may arise from mechanical interactions between prosthetic components (e.g., between the femoral component and tibial insert), soft tissue tension (such as clunk syndrome or snapping phenomena) [27,28] or postoperative changes in the patellofemoral joint (e.g., patellar grinding). Implant malposition, joint instability, and patellofemoral maltracking are also recognized contributors to noise generation, highlighting the importance of accurate component positioning [29,30,31].

In cases where the noise occurrence is not constant and is not associated with impaired function, patients can be offered reassurance, general advice around modifying activities and close monitoring in the follow-up period. The majority of patients with noisy TKA do not require further surgical intervention. However, in rare cases where there is persistent or troublesome noise, surgery may be required. Through soft tissue tension optimization, correction of any tissue/bone impingement or component malposition, improvements can be achieved.

The present study found that, although functional outcomes did not differ significantly between patients who reported prosthetic noise and those who did not, patient satisfaction was significantly lower in individuals experiencing noise. This association suggests that patients reporting prosthetic noise also report lower satisfaction, although the observational retrospective design of the study does not allow a determination of whether prosthetic noise independently contributes to dissatisfaction. These findings contrast with those of Taniguchi et al. [14], who reported that noise perception after TKA had a limited effect on joint awareness and patient-reported clinical outcomes. This discrepancy may be explained by differences in outcome measures, study populations, or the way noise perception was assessed. While Taniguchi et al. [14] focused primarily on joint awareness and functional PROMs, patient satisfaction represents a broader and more complex construct that encompasses psychological and emotional factors in addition to physical function. It is therefore plausible that prosthetic noise, although generally benign, may generate concern or dissatisfaction by being perceived as abnormal or suggestive of implant dysfunction by patients. This highlights the importance of considering not only objective functional outcomes but also subjective patient perceptions when evaluating the success of TKA. In this context, preoperative patient education and appropriate counseling regarding the possible occurrence and generally benign nature of prosthetic noise may help mitigate its negative impact on postoperative satisfaction. While the presence of prosthetic noise does not typically indicate mechanical failure or clinical complications, it may have a significant psychological impact on patients [32]. Perceived noise can lead to concern, distress, or dissatisfaction, particularly when interpreted as a sign of implant dysfunction or damage. Although native degenerative knees may also produce noise, the postoperative context often amplifies patient awareness, leading to a negative association with the prosthesis.

This highlights the importance of the psychological dimension in postoperative recovery and underscores the need for appropriate patient education. Patient-related factors may also contribute to prosthetic noise generation. Younger age has been identified as a potential risk factor, as demonstrated by Lizcano et al. [23], with younger patients more likely to report noise following TKA [33]. This may be explained by greater activity levels and heightened sensory perception in this population. Notably, prosthetic noise tends to decrease over time [34], likely reflecting progressive adaptation of the implant–soft tissue interface and improved biomechanical stability as the components (femoral component, tibial insert, and patella) settle during functional activities. In addition, postoperative swelling may exacerbate noise perception, with patients often reporting improvement as joint effusion resolves. Taken together, these findings highlight the multifactorial nature of prosthetic noise and suggest that both patient-specific and temporal factors should be considered. In this context, prosthetic design may still play a role and could be considered as part of the decision-making process when discussing TKA options with patients [35,36].

This study has several limitations. The principal limitation is the absence of radiographic evaluation. Because prosthetic noise may be related to component malposition, malalignment, instability, patellofemoral maltracking, or other structural factors, the lack of standardized radiographic assessment prevented investigation of potential mechanical causes of noise generation. This limitation is particularly important given the observed association between prosthetic noise and lower SKV scores. Patient assessment was also conducted by telephone rather than through in-person clinical evaluation, precluding objective physical examination. In addition, the retrospective and non-randomized design introduces potential selection bias, while unmeasured confounding variables such as BMI, range of motion, activity level, and preoperative deformity may have influenced the results. Also, no a priori sample size calculation was performed because of the retrospective design of the study. Consequently, the study may have been underpowered to detect small but clinically relevant differences in prosthetic noise between implant designs. Therefore, the absence of a statistically significant difference should not be interpreted as evidence of equivalence, and larger prospective studies are warranted to confirm these findings. Finally, several outcomes relied on patient-reported measures and were therefore inherently subjective.

Future prospective randomized studies including potential confounding variables are needed to better determine the independent effect of implant design on prosthetic noise after TKA and to better delineate the relative contributions of surgical technique, implant design, and patient-specific factors in noise generation.

## 5. Conclusions

In this retrospective comparative study, no statistically significant difference in prosthetic noise was detected between mobile-bearing and cruciate-retaining total knee arthroplasty designs. Patients reporting prosthetic noise had lower satisfaction, whereas functional outcomes were comparable. Given the retrospective design and the absence of adjustment for potential confounding factors, these findings should be interpreted with caution. Larger prospective studies are needed to confirm these observations and to better define the influence of implant design on prosthetic noise and patient satisfaction.

## Figures and Tables

**Table 1:** Comparable demographic data in the two groups.

Variable	Score^®^ Amplitude MB (*n* = 94)	Stryker Triathlon^®^ CR (*n* = 118)	Cohort Analysis (*n* = 212)	*p*-Value
Age, mean (SD)	70.1 (8.9)	68.8 (8.0)	68.3 (8.3)	0.20
Age, median (Q1; Q3)	71.5 (64.5; 76.5)	68.0 (63.0; 75.0)	69.0 (62.0; 75.0)	
Age, min–max	42–87	52–86	42–87	
<65 years, n (%)	24 (25.5)	37 (31.4)	61 (28.8)	
65–69 years, n (%)	19 (20.2)	28 (23.7)	47 (22.2)	
70–74 years, n (%)	15 (16.0)	20 (16.9)	35 (16.5)	
≥75 years, n (%)	36 (38.3)	33 (28.0)	69 (32.5)	
Sex				0.8526
Male, n (%)	49 (52.1)	67 (56.8)	116 (54.7)	
Female, n (%)	45 (47.9)	51 (43.2)	96 (45.3)	

SD: standard deviation; Q1: first quartile; Q3: third quartile.

**Table 2:** Values of functional scores (SKV and IKS) in the two groups (comparable).

	Score^®^ Amplitude MB	Stryker Triathlon^®^ CR	*p*-Value
**Simple Knee Value (SKV),**			
Mean (SD)	81.5 (13.2)	77.4 (15.5)	0.4121 (ANOVA)
Median (Q1; Q3)	80.0 (80.0; 90.0)	80.0 (70.0; 90.0)	
Min;Max	[35;100]	[40;100]	
**International Knee Score (IKS),**			
Mean (SD)	35.6 (6.6)	34.6 (6.8)	0.6927 (ANOVA)
Median (Q1; Q3)	38.0 (35.0; 40.0)	37.0 (34.0; 40.0)	
Min;Max	[10;40]	[14;40]	

SD: Standard deviation; Q1: 1st Quartile; Q3: 3rd Quartile.

## Data Availability

The data presented in this study are available on request from the corresponding author.
